# Ultrasound Prenatal Diagnosis of Inguinal Scrotal Hernia and Contralateral Hydrocele

**DOI:** 10.1155/2013/764579

**Published:** 2013-12-12

**Authors:** G. Massaro, G. Sglavo, A. Cavallaro, G. Pastore, C. Nappi, C. Di Carlo

**Affiliations:** Department of Obstetrics and Gynecology, University of Naples Federico II, Via Pansini 5, 80131 Naples, Italy

## Abstract

Fetal inguinal scrotal hernia is a rare condition resulting in an abnormal embryonic process of the tunica vaginalis. We report a case of ultrasound prenatal diagnosis of inguinal scrotal hernia associated with contralateral hydrocele in a woman at 37 weeks of gestation, referred to our clinic for a scrotal mass. Differential diagnosis includes hydrocele, teratoma, hemangiomas, solid tumours of testis, bowel herniation, and testicular torsion. Bowel peristalsis is an important ultrasound sign and it allowed us to make diagnosis of inguinal scrotal hernia. Diagnosis was confirmed at birth and a laparoscopic hernia repair was performed without complications on day 10. During surgery, a bilateral defect of canal inguinal was seen and considered as the cause of scrotal inguinal hernia and contralateral hydrocele observed in utero.

## 1. Introduction

Inguinal hernia is found in 1–4% of neonates and children. It is more common in male and is usually an isolated finding.

Pediatric inguinal hernias and hydroceles are due to incomplete or abnormal obliteration of the processus vaginalis peritonei [1–3]. This is a tubular fold of the peritoneum that invaginates into the inguinal canal anterior to the gubernaculum and descending testis, ending in the scrotum. The upper part usually closes at or just before birth, and obliteration proceeds gradually in a downward direction. The scrotal section remains patent, forming the tunica vaginalis testis [2].

If the canal does not close, at birth, the increasing intra-abdominal pressure can force bowel loops into the scrotum determining inguinal hernia, which is a common pediatric disease [1, 2].

Prenatal inguinal hernia is a rare condition as intra-abdominal pressure in the fetus is similar to the pressure in the amniotic cavity. Therefore very few cases are described in the literature.

In this paper we report a case of prenatal inguinal scrotal hernia, associated with contralateral hydrocele, diagnosed by ultrasound examination at 37 weeks of gestation.

## 2. Case Report

A 30-year old woman, primigravida, was referred to the Fetal Medicine Clinic of our department at 37 weeks of gestation for evaluation of a fetal scrotal mass visualized on ultrasound. Previous obstetric ultrasound examinations at 12, 21, and 30 weeks had not shown any evident fetal abnormalities. Fetal karyotyping had not been performed.

The ultrasound examination was performed using a 4.0–8.0 MHz multifrequency 3D transducer (Voluson 730 Expert, General Electric). Fetal biometry was within the normal ranges for the gestational age with normal biparietal diameter and head circumference and femur length and abdomen circumference. The amount of amniotic fluid was in the normal range.

**Figure 1:**
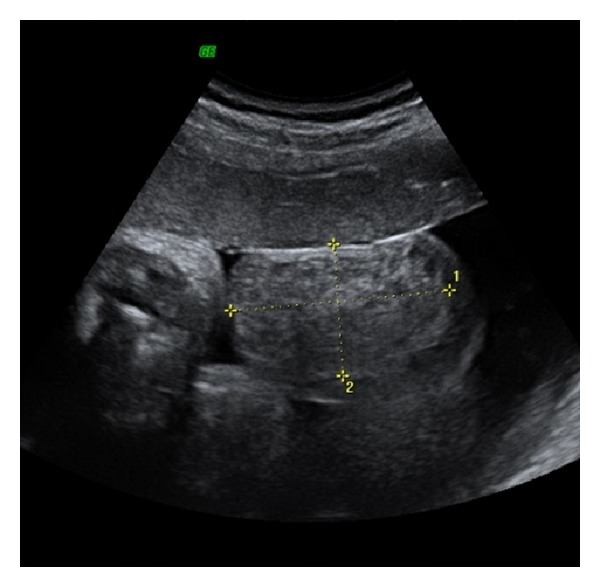
Echogenic scrotal mass (50 × 46 mm) with mixed echostructure and regular walls, containing few small echo-free cystic areas, in the right side.

**Figure 2:**
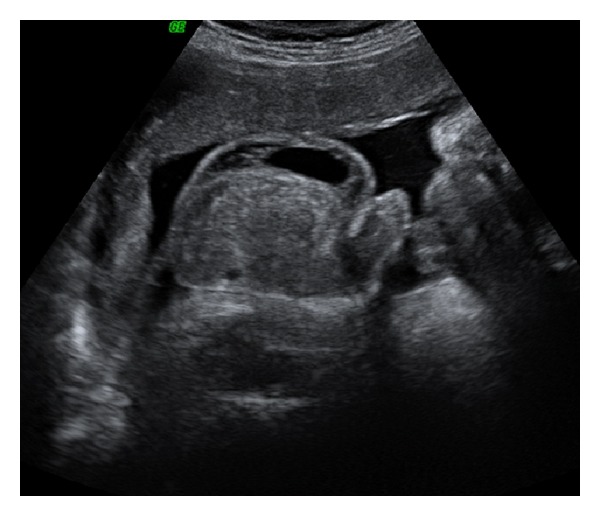
Hydrocele in the left scrotal side.

The study of the scrotum revealed an echogenic mass (50 × 46 mm) with mixed echostructure and regular walls, containing few small echo-free cystic areas in the right side (Figure 1) and hydrocele in the contralateral side (Figure 2). During the examination, no intestinal peristalsis was seen in the scrotal mass and there was no evidence of an associated bowel obstruction or meconium peritonitis. No other fetal abnormalities were found. Color Doppler examination showed the presence of regular vessels in the testicular sac.

A pediatric surgeon counselling was requested after our examination. The consultant advised for clinical evaluation at birth, and emergency surgery in case of complications.

**Figure 3:**
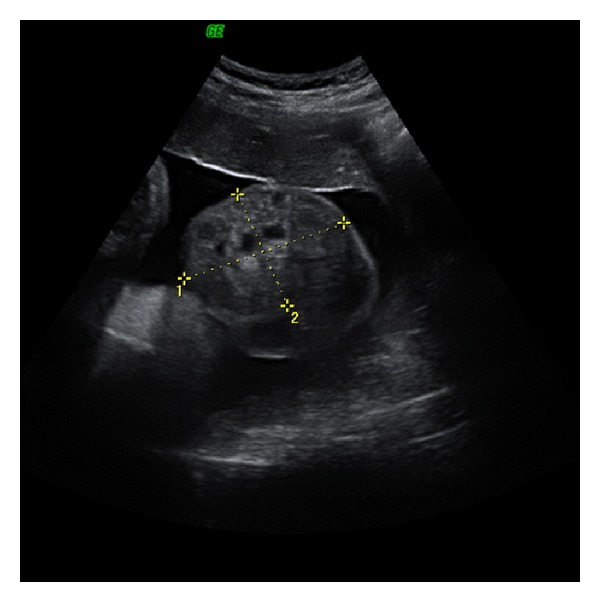
A week later: no modification in size of the mass; an increasing number of echo-free cystic areas.

The following ultrasound examination, performed a week later, found no modification in size of the mass and an increasing number of echo-free cystic areas, without signs of complications (Figure 3). In addition, bowel peristalsis was observed in the scrotal mass allowing us to make diagnoses of inguinal scrotal hernia.

A caesarean section was performed with no complications at 38w + 6d, for podalic presentation.

**Figure 4:**
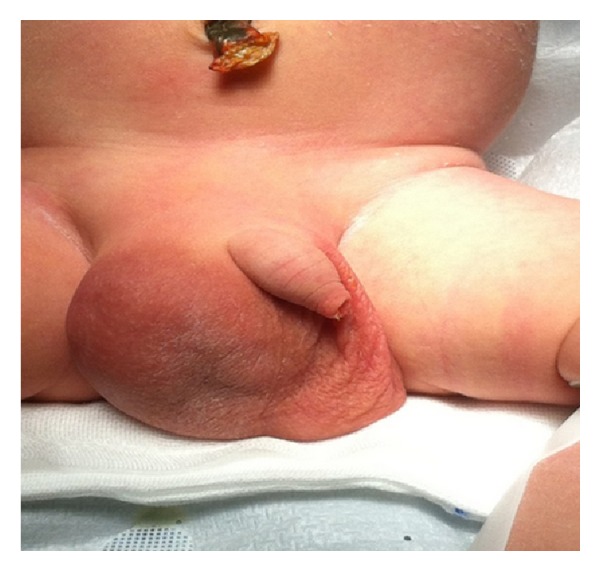
The presence of right inguinal scrotal hernia at birth.

The neonate weight was 3700 g with an Apgar score of 8 and 9 at 1 and 5 minutes, respectively. The pediatric examination confirmed the presence of right inguinal scrotal hernia (Figure 4).

**Figure 5:**
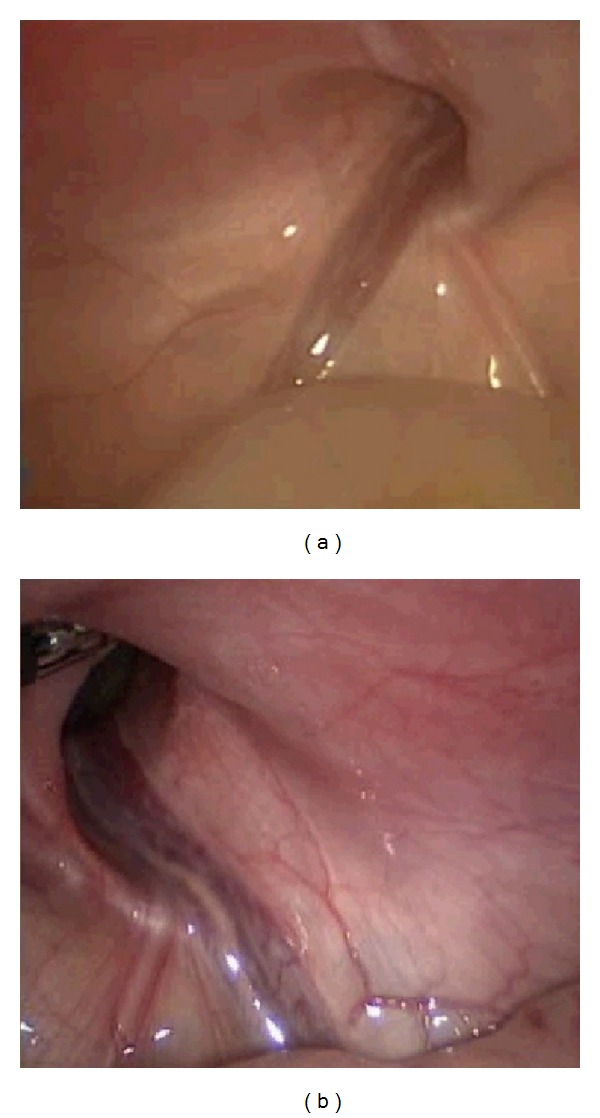
Laparoscopic repair of bilateral defect of the inguinal canal.

The neonate was dismissed on the fourth day after birth and was admitted in the Pediatric Surgery Department of our hospital, where, on day 10, a laparoscopic hernia repair was performed without complications. During surgery, a similar contralateral defect of the inguinal canal was observed and repaired. This was the cause of the left hydrocele, seen in utero (Figure 5).

No bowel symptoms on follow-up examination at 3 months were found.

## 3. Discussion

The prenatal diagnosis of inguinal scrotal hernia is a rare occurrence, and very few cases are described in the literature [4–14].

Differential diagnosis of scrotal mass includes hydrocele, teratoma, hemangiomas, solid tumours of testis, bowel herniation, and testicular torsion.

The most common cause of scrotal mass is *hydrocele*; it is characterized by a fluid-filled space within the scrotum next to the testes; sonographic features are given by a typical “half moon” fluid image surrounding the testis [1, 7].

Less common is *teratoma testis*; it appears as a mixed echostructure mass, usually larger and highly vascularised on color Doppler examination (evidence of vascularity and flow) [15–17].

*Testicular torsion* in a male fetus is characterized by an enlarged testis and epididymis and accumulation of hemorrhagic fluid between the visceral and parietal layers of the tunica vaginalis and outside the tunica vaginalis, resulting in “double-ring hemorrhage” image; in addition it is associated with the absence of testicular flow on color Doppler examination [18, 19].

Clinical and sonographic characteristics of *fetal inguinal scrotal hernia* include the following: maternal gestational age not less than 26 weeks; scrotal mass with smooth contour and predominant solid content with internal echo-free cystic components, consistent with bowel loops; intestinal peristalsis within the scrotum at real-time sonography, with movement and deformation of the echo-free cystic components [4–11]. Furthermore, color Doppler examination shows the presence of vessels in the testis originating from the mesenteric artery; this feature demonstrates that the mass is an inguinal scrotal hernia without strangulation [11]. Signs of inguinal scrotal hernia with strangulation and bowel obstruction are the absence of intestinal peristalsis and the presence of multiple dilated bowel loops and polyhydramnios [8].

In our case, at first examination, we found a smooth contour mass with small echo-free cystic components and no bowel peristalses; in the contralateral scrotal region we observed hydrocele. At this time, other possible diagnoses were considered and an ultrasound control was prescribed after one week when we found a more clear visualization of the echo-free/cystic areas and the presence of bowel peristalsis, allowing us to diagnose inguinal scrotal hernia [8].

In conclusion, when a scrotal mass is identified in utero, it is important to evaluate all ultrasonographic signs of the lesion to discriminate inguinal scrotal hernia from teratoma, hydrocele, or testicular torsion so as to have a correct prognosis and avoid pre- and postnatal complication.
